# Prevalence and spatial distribution of cranberry fruit rot pathogens in British Columbia, Canada and potential fungicides for fruit rot management

**DOI:** 10.3389/fpls.2023.1274094

**Published:** 2023-11-10

**Authors:** Brandon Wood, Ethan McBride, Keiko Nabetani, Taylor Griffin, Siva Sabaratnam

**Affiliations:** ^1^ Abbotsford Agriculture Centre, Ministry of Agriculture and Food, Abbotsford, BC, Canada; ^2^ Reconciliation, Partnerships and Indigenous Fisheries, Fisheries and Oceans Canada, Vancouver, BC, Canada; ^3^ Crop Development Centre, Plant Sciences, University of Saskatchewan, Saskatoon, SK, Canada

**Keywords:** fruit rot, fungal pathogens, prevalence, incidence, spatial distribution, fungicides, fungicide efficacy

## Abstract

Twenty-eight cranberry farms in southwestern British Columbia were investigated for the prevalence and spatial distribution of fungal pathogens that contribute to fruit rot incidence. Farms were selected from six regions where most cranberry production is concentrated. Flowers, and green and ripe fruit (var. ‘Stevens’) samples, collected during two consecutive crop seasons, were analyzed for fruit rot pathogens. The most frequently isolated pathogens were identified as *Allantophomopsis cytisporea*, *Botrytis cinerea*, *Coleophoma empetri*, *Colletotrichum fioriniae*, *Colletotrichum gloeosporioides*, *Fusicoccum putrefaciens*, *Glomerella* sp., *Phomopsis vaccinii*, *Phyllosticta elongata*, *Phyllosticta vaccinii* and *Physalospora vaccinii*. The pathogens *Allantophomopsis cytisporea*, *Phyllosticta* spp., and *Physalospora vaccinii* were found at high incidence. These pathogens were present in all cranberry growing regions, although their mean percentage incidence varied from farm-to-farm and region-to-region. Amongst the pathogens from three phenological stages of cranberry crop examined, ripe fruit had the highest percentage incidence of fruit rot pathogens compared to that of flowers or green fruit; thus, indicating their presence at the early stages of crop development. The efficacy to inhibit the mycelial growth and spore germination of the fruit rot pathogens by twenty six fungicides, belonging to nine different modes of actions, were evaluated *in vitro*. The copper-based fungicides and captan of group M, flutriafol, triforine, difenoconazole, prothioconazole and propiconazole of group 3, benzovindiflupyr of group 7, and fosetyl-Al of group 33 demonstrated a high degree of efficacy in inhibiting the mycelial growth of all fruit rot pathogens. The fungicides chlorothalonil of group M, fenbuconazole of group 3, pyrimethanil and cyprodinil of group 9, and fludioxonil of group 12 also demonstrated activity against most fruit rot pathogens. The copper-based fungicides, chlorothalonil, captan, flutriafol, triforine, difenoconazole, prothioconazole, propiconazole, benzovindiflupyr, and fosetyl-Al effectively prevented the spore germination of most fruit rot pathogens. This demonstrated activity of the fungicides towards cranberry fruit rot pathogens should be assessed for efficacy *in planta* under field conditions. The current study identified the most critical fungal pathogens causing fruit rot of cranberry in British Columbia and potential fungicides that could be used in the management of fruit rot and to improve fruit quality and yield.

## 1 Introduction

British Columbia is the second largest producer of cranberry (*Vaccinium macrocarpon*) in Canada with an annual farm gate value of over $42 million CAN. (British Columbia Cranberry Industry Snapshot, 2017; Statistics Canada, 2020). In British Columbia, cranberry is cultivated on 2630 hectares of agricultural land, mostly concentrated in the southwestern region (Lower Mainland) and on Vancouver Island. British Columbia’s cranberry production is accounted for approximately 29% of the Canadian and 12% of the North America markets (Crop Profile for Cranberry in Canada, 2019; British Columbia Cranberry Marketing Commission, 2021). In North America, almost 70% of the production is in the northeastern regions of the United States, mostly in Wisconsin, Massachusetts, and New Jersey (Roper, 2006; Eklund, 2014). Regardless of where it is being cultivated, pre- and post-harvest fruit rot caused by fungal pathogens have significantly impacted fruit yield and quality ever since the first commercial fields were established in North America well over a hundred years ago (Roper and Vorsa, 1997; Oudemans et al., 1998). Cranberry fruit rot is considered as a ‘disease complex’ caused by several fungal pathogens. However, the term ‘fruit rot’ has been widely used to describe the disease complex as a single entity due to difficulties in distinguishing the differences in the symptoms caused by the pathogens, and the complexity in identifying the pathogens involved (Oudemans et al., 1998; Olatinwo et al., 2003; Robideau et al., 2008; Tadych et al., 2012).

Several fungal pathogens have been known to cause fruit rot of cranberries, primarily *Allantophomopsis lycopodina* (Höhn.) Carris, *Allantophomopsis cytisporea* (Fr.) Petr. (syn. *Ceuthospora lunata* and *Apostrasseria lunata*), *Colletotrichum fioriniae* Marcelino & Gouli ex. R.G. Shivas & Y.P. Tan or formally *Colletotrichum acutatum* (teleomorph *Glomerella acutata*), *Colletotrichum gloeosporioides* (Penz.) Penz. & Sacc., including *Colletotrichum fructivorum* (teleomorph *Glomerella cingulata*), *Coleophoma empetri* Rostr. (Syn. *Sporonema oxycocci*), *Fusicoccum putrefaciens* Shear (teleomorph *Godronia cassandrae* f. *vaccinii* (syn.), *Physalospora vaccinii* (shear) Arx & E. Müll., *Phyllosticta vaccinii* (Earle), *Phyllosticta elongata* (syn. *Phyllosticta vaccinii*), *Phomopsis vaccinii* Shear (teleomorph *Diaporthe vaccinii*), and *Botrytis* spp. (Zuckerman, 1958; Stiles and Oudemans, 1999; Olatinwo et al., 2003; Polashock et al., 2009; Polashock et al., 2017; Wells-Hansen and McManus, 2017; Patten and Daniels, 2019; Conti et al., 2022). Although there is a general understanding of the fungal pathogens associated with cranberry fruit rot, their prevalence, incidence and spatial distribution in British Columbia have not been investigated previously. Over the years, farmers in British Columbia have adopted the management strategies for the fruit rot complex employed by farmers in the northeastern United States. However, it is expected that the qualitative and quantitative nature of the fungal pathogens associated with the fruit rot complex in the Pacific Northwest, including British Columbia, vary from those present in the cranberry growing regions of northeastern North America. This could be due to the geographical separation, differences in the climatic conditions and cultivation practices between the cranberry growing regions in the eastern and western North America. As an example of different cultivation practices, in Massachusetts and Wisconsin, cranberry is cultivated on a sand-based soil where the top layer of peat or organic material is replaced with a layer of 10 to 30 cm of sand at pre-planting. An additional 2 to 10 cm layer of sand is applied every two to five years to suppress weeds and insect populations and to encourage root health and canopy growth (Roper, 2007; Averill et al., 2008; Colquhoun and Johnson, 2020). There is anecdotal evidence that sand-based fields tend to suppress plant pathogens (Oudemans et al., 1998; Averill et al., 2008). In British Columbia, cranberry is predominantly grown on peat- or muck-based soil without the amendment of sand, although a small percentage of the fields are mineral soils where farmers choose to apply either sawdust or sandy gravel as a top layer (Strik et al., 2002). In British Columbia, besides the differences in the growing conditions, there are noticeable differences in cultural practices, including irrigation methods, types of fungicides, their use pattern and application methods, and harvesting practices. In Wisconsin, flooding the cranberry fields during winter months is a common practice to prevent winter damage. In Massachusetts, flooding for two to three weeks prior to spring has been shown to suppress weeds and certain insect pests, and to a lesser extent fungal pathogens (Averill et al., 1997). In Wisconsin, it is speculated that the delayed blooming of the cranberry crop triggered by flooding practices aids the crop to overcome the critical times of infection by some fungal pathogens (McManus, 2001); however, such practices have shown poor success in New Jersey (Averill et al., 2008). Flooding has not been adopted by the farmers in British Columbia, except when fields are flooded for three to five days in late September to early November for harvesting fruit (personal communication). These differences in the overall production practices amongst the cranberry producing regions in North America can contribute to the differences in the prevalence, incidence, and spatial distribution of fruit rot pathogens. As a result, their impact on fruit quality and yield is expected to vary from region to region; thus, emphasizing the need for understanding the diversity of fungal pathogens and their impact on cranberry production in each region, including British Columbia.

Use of fungicides to control fruit rot pathogens is an integral part of the overall best management practices employed by cranberry farmers in British Columbia. However, the number of fungicides available to farmers in British Columbia is far less than their counterparts in the USA. It is primarily due to the differences in the federal regulatory guidelines and fungicide application technology between the two countries and cranberry producing regions. In British Columbia, pesticides, including fungicides, are applied to cranberry fields through the irrigation system, referred to as ‘chemigation’. Therefore, the province has to meet specific regulatory requirements and environmental safety measures in order to be eligible for the approval of pesticides by the government of Canada. Furthermore, the complex nature of fungal pathogens associated with fruit rot and not knowing their presence or overall contribution to fruit loss make it difficult to select the most effective fungicides to specific fruit rot pathogens. In this study, an attempt was made to evaluate a wide spectrum of fungicides with different modes of actions in accordance with the international Fungicide Resistance Action Committee guidelines (FRAC Code List, 2022).

The objectives of this study were to a) identify and characterize the major fungal pathogens of the cranberry fruit rot complex in British Columbia, b) understand the incidence and spatial distribution of fruit rot pathogens amongst different cranberry growing regions, c) assess the overall impact of fruit rot pathogens on cranberry production, and d) evaluate the efficacy of fungicides for inhibition of growth of mycelium and spore germination of cranberry fruit rot pathogens. The knowledge gathered from the studies will identify the major fruit rot pathogens that are present in cranberry farms and support the development of management strategies for controlling the cranberry fruit rot complex, and, thus, improve fruit quality and yield of cranberry production in British Columbia.

## 2 Materials and methods

### 2.1 Prevalence, incidence and spatial distribution of cranberry fruit rot fungi

**Figure 1:**
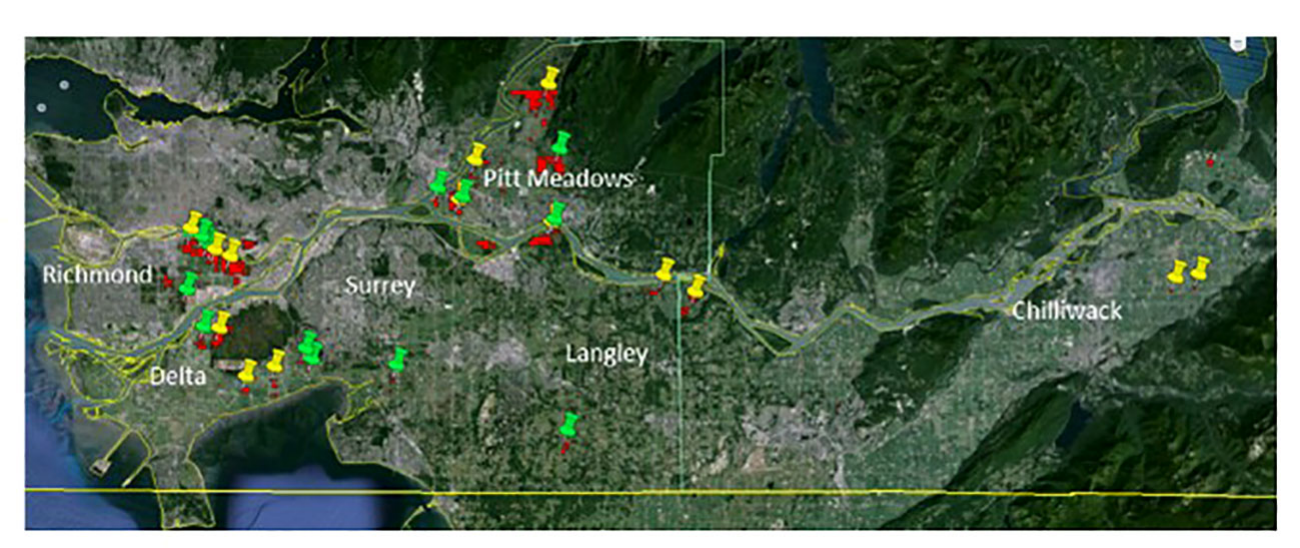
Geographical locations of the twenty-eight cranberry farms in southwestern British Columbia (Lower Mainland) that were sampled in two consecutive years (Year 1 - yellow targets, and Year 2 - green targets). Samples of flowers, and green and ripe fruits were collected from the farms and analyzed for the prevalence and spatial distribution of cranberry fruit rot pathogens.

Cranberry farms in different geographical regions of southwestern British Columbia (Lower Mainland) were surveyed and sampled during two consecutive crop seasons of 2016 and 2017, hereinafter referred to as Year 1 and Year 2, respectively, to understand the prevalence, incidence and spatial distribution of fungal pathogens contributing to the fruit rot complex. In both years, a total of twenty-eight nonorganic, commercial farms (twenty to thirty years old) in six regions, Chilliwack, Langley, Surrey, Pitt Meadows, Richmond and Delta were included in the study (**Figure 1**). Cultivar ‘Stevens’ was chosen to ensure consistency in the sampling as it is the most commonly grown cultivar in British Columbia. Samples were collected at three phenological stages of the cranberry crop, flowers at 50% in-bloom, green fruit at four to six weeks of fruit-set, and ripe fruit at harvest. In each field, three 2 m^2^ sampling blocks were selected as replicates and spaced diagonally from one corner to the centre of the field. At each sampling, a sample set of either twenty flowers, twenty green or twenty ripe fruits were randomly collected along the 2 diagonal lines of a 1 m^2^ transect placed within each sampling block; a new sampling area within the 2 m^2^ block was used for each of the three samplings. Samples were collected using a pair of sterile forceps and placed in re-sealable plastic bags in a cooler box with icepacks for transporting to the laboratory. The samples were kept at 4°C and processed the following day.

#### 2.1.1 Isolation of fruit rot fungi

The sixty flowers, green fruit or ripe fruit collected from each of twenty-eight farms were processed within 48 h of sampling. Samples were prepared by removing the peduncle of the flowers and berries with a pair of sterile tweezers. Flowers and fruits were surface sterilized in a 0.5% NaClO solution supplemented with 0.1% TWEEN^®^ 20 (Sigma-Aldrich) for 1 min under a gentile agitation on a magnetic shaker and then rinsed repeatedly three times with sterile distilled water. Excess moisture on the flower and fruit surfaces was removed by placing them on a sterile paper towel inside a laminar flow chamber. The flowers and fruits were cut longitudinally into two-halves with a sterile scalpel. The dissected halves of the flowers and fruits were placed by facing the cut-end down on acidified quarter-strength potato-dextrose-agar medium (a¼PDA) (Difco Laboratories, USA) in 90 cm Petri dishes. Acidified medium was prepared by supplementing 2 mL of 25% lactic acid to 998 mL of ¼PDA. The Petri dishes with the cranberry tissues were incubated in the dark at 22°C and examined periodically for the growth of fungal colonies originating from the tissues.

#### 2.2.2 Prevalence, incidence and spatial distribution

The fungal colonies that originated from the samples of flowers, and green and ripe fruits on a¼PDA medium were transferred to ¼ PDA and PDA media and grown at 20-24°C in the dark for at least two to four weeks until they produced spores in culture. Isolates that failed to produce spores were exposed to 14 h UV-A (320-400 nm) and a 10 h dark photoperiod at 22°C to further induce sporulation (Leach, 1962; Dahlberg and Etten, 1982). Based on the colony characteristics and morphology of spores produced on culture media, fungal isolates were tentatively identified to their genus or, in some cases, species (Wells and McManus, 2013; Polashock et al., 2017; CMI Description of Pathogenic Fungi and Bacteria, 1969; Polashock et al., 2009). Based on the initial identification, a subset of representative isolates was further characterized using DNA-based molecular markers. Pure cultures of the fungal isolates generated either from a single spore or hyphal tip of the isolates (Choi et al., 1999) were grown in a sterile ¼PD broth medium at ambient temperature at 70 RPM on an orbital shaker (Thermolyne™ AROS 160) for three to five days. The DNA of mycelium from the broth cultures were extracted using a DNeasy Plant Mini Kit (QIAGEN Inc., Toronto, ON, Canada), eluted in 100 µl buffer AE (QIAGEN Inc.) and stored at -20°C. The internally transcribed spacers of 5.8S nuclear rDNA were amplified with the general fungal nucleotide primers, ITS1 and ITS4 (White et al., 1990). Amplification of DNA was performed in a PCR System (ProFlex™; Life Technologies Inc., Burlington, ON, Canada), using illustra PuReTaq Ready-To-Go™ PCR Beads (GE Healthcare Life Sciences, Mississauga, ON, Canada) in a total volume of 25 µL, containing 21 µL of sterile distilled water, 40 µM of each primer, and 2 µL of the extracted DNA of the fungal isolate. PCR thermal cycle conditions were set as follows: initial denaturation at 94°C for 2 min, followed by thirty five cycles for 30 sec at 94°C, 55°C for 40 sec, 72°C for 60 sec, and final extension at 72°C for 10 min. Amplified DNA was confirmed with agarose gel electrophoresis, purified using Amicon™Ultra 0.5 ml centrifugal filters with Ultracel™ 30K membranes (Millipore Canada Ltd., Etobicoke, ON, Canada), and subjected to bidirectional sequencing (Bio Basic Canada Inc., Markham, ON, Canada). DNA sequences were compared (aligned) with GenBank nucleotide sequences by BLAST (Basic Local Alignment Search Tool) analysis to provide consensus for the identification of the fungal isolates. Based on the molecular characterization, the fungal isolates were identified to their genus and species. Pure cultures of the fungal isolates representing each species were grown on PDA medium for seven to fourteen days, photographed and documented. Based on the literature and identity of the fungal pathogens obtained from the cranberry tissues, the frequency of each of the pathogens found on flowers, green and ripe fruits from the twenty-eight farms was recorded. The data were used to determine the most prevalent fruit rot pathogens present in cranberry fields in British Columbia, their percentage incidence at flowering, and green and ripe fruit stages in each farm, and their spatial distribution amongst the different cranberry growing regions in southwestern British Columbia.

#### 2.1.3 Assessment of fruit rot incidence

To assess the percentage incidence of fruit loss due to fruit rot caused by fungal pathogens in cranberry farms, a subset of samples of ripe fruit was collected just prior to harvest. Fruit samples were collected from a total of twenty-eight cranberry farms during two consecutive crop seasons by following a similar sampling protocol as described previously. From each field, at least forty ripe fruit were randomly collected along the two diagonal lines of a 1 m^2^ transect placed within each of three 2 m^2^ sampling plots; thus, a total of one hundred and twenty fruits were collected from each field. Fruit samples were placed in re-sealable plastic bags, kept in a cooler box with icepacks and brought to the laboratory. The samples were kept at 4°C and processed the following day. Fruits collected from each farm were examined visually with a hand-held lens (x10) for signs of visible symptoms, and the healthy and symptomatic fruits were separated, counted and recorded. Fruits were considered symptomatic based on the change of colour, shape, texture or necrotic lesions on fruit that differed from the healthy fruit. Healthy fruit were placed in a humid chamber for 3 weeks at ambient temperature (~24°C) and assessed for any fruit rot symptoms as described above. A three-week holding was chosen to account for the time spent in the event of delayed harvest, transportation, grading and packing of harvested fruit in the processing and packing facilities (personal communication, Ocean Spray, Richmond, B.C.). Wherever possible, pathogens present on the symptomatic fruit at three-week incubation were identified based on spore morphology or by culturing them on a½PDA and PDA media as described previously. The percentage incidence of fruit-rot at harvest (i.e., field rot) and three-week incubation (i.e., post-harvest or storage rot), and the cumulative percentage of total fruit loss in each farm were calculated.

### 2.2 Efficacy of fungicides to fruit rot pathogens

A total of twenty six chemical fungicides, belonging to nine different groups of modes of actions (FRAC Code List, 2022), were evaluated *in vitro* for their efficacy to inhibit mycelial growth and spore germination of the major cranberry fruit rot pathogens as identified previously. Fungicides were tested at the minimum and maximum application rates as per the manufacturers’ label recommendation (**Table 1**). In order to avoid discrepancy in the application rates amongst different fungicides, minimum and maximum application rates of each fungicide (either by weight or volume of the fungicide per hectare or per spray volume) were calculated in 500 L water as a standard volume that would mimic the chemigation of fungicides to cranberry fields in British Columbia. For each pathogen, at least two or three isolates representing different geographical regions, i.e., a total of twenty three isolates of ten major pathogens, were included in the study. For the genus *Colletotrichum*, 3 isolates from each of the three species, *C*. *acutatum*, *C*. *gloeosporioides* and *Glomerella cingulata* (teleomorph of *Colletotrichum* spp.) were included. For *Physalospora vaccinii*, two isolates of each of the dark and light-coloured phenotypes were included.

**Table 1:** Twenty-six chemical fungicides, belonging to 9 different groups of modes of actions (FRAC Code List, 2022), were evaluated at the minimum and maximum application rates for the efficacy to inhibit the mycelial growth of cranberry fruit rot pathogens.

Inhibition of mycelial growth (scale of 1 to 10) of cranberry fruit rot pathogens by fungicides																							
Fungicide (active ingredient)				Fruit rot pathogens																			
				*A. lycopodina*		*B. cinerea*		*C. empetri*		*C. acutatum*		*C. gloeosporioides*		*F. putrefaciens*		*G. cingulata*		*Phom. vaccinii*		*Phyl. elongata*		*Phys. vaccinii*	
Fungicide Concentration (ppm) :		Min.	Max.	Mi.	Mx.	Mi.	Mx.	Mi.	Mx.	Mi.	Mx.	Mi.	Mx.	Mi.	Mx.	Mi.	Mx.	Mi.	Mx.	Mi.	Mx.	Mi.	Mx.
Group M	Bravo (chlorothalonil)	6800	11600	8	8	7	7	6	5	8	8	8	8	4	3	8	8	9	8	8	8	8	8
	Copper 53W (copper sulfate)	3204	5340	10	10	10	9	10	10	10	10	10	10	8	10	10	10	10	10	10	10	10	10
	Cueva (copper octanoate)	90	360	10	9	10	10	10	10	10	10	10	10	8	9	10	10	10	10	10	10	10	10
	Guardsman(copper oxychloride)	2000	4000	8	9	6	6	10	8	8	8	8	8	3	3	8	8	8	8	10	10	10	10
	Kocide (copper hydroxide)	2229	4458	10	10	8	8	10	10	10	10	9	10	9	10	8	9	10	10	10	10	10	10
	Maestro (captan)	4400	6800	10	10	8	8	8	6	8	8	9	8	8	8	9	9	9	9	10	10	10	10
Group 3	Fullback (flutriafol)	128	256	10	10	10	10	10	10	9	9	9	9	10	10	10	10	10	10	10	10	10	10
	Funginex (triforine)	570	1140	10	10	10	9	10	10	10	10	10	10	9	10	9	10	10	10	10	10	10	10
	Indar (fenbuconazole)	105	210	10	10	9	9	10	10	8	8	7	7	10	10	6	7	10	10	9	9	7	7
	Inspire (difenoconazole)	73	145	10	10	9	9	10	10	9	10	9	9	8	10	9	10	10	10	10	9	10	10
	Proline (prothioconazole)	175	350	10	10	9	10	10	10	10	10	10	10	10	10	10	10	10	10	10	10	10	10
	Tilt (propiconazole)	125	250	10	10	9	9	10	10	10	10	10	10	10	10	10	10	10	10	10	10	10	10
Group 7	Adepidyn (pydiflumetofen)	149	299	4	4	6	7	6	5	4	4	3	3	5	5	5	4	5	6	8	10	4	4
	Aprovia (benzovindiflupyr)	100	150	9	10	9	9	10	10	8	9	8	8	10	10	9	10	10	9	10	10	10	10
	Fontelis (penthiopyrad)	400	700	3	3	4	5	2	3	7	7	7	7	2	3	9	9	4	4	7	7	3	3
	Kenja (isofetamid)	790	992	3	2	5	5	1	2	1	1	1	1	3	2	4	4	4	4	7	7	2	2
	Sercadis (fluxapyroxad)	150	400	2	3	6	6	3	3	1	1	2	3	1	1	4	4	5	4	6	7	2	2
Group 9	Scala (pyrimethanil)	800	1600	8	9	9	10	10	10	6	7	7	7	10	10	8	8	9	9	10	10	10	10
	Vangard (cyprodinil)	563	1125	8	9	9	9	6	6	5	5	5	5	8	8	7	6	8	8	9	9	9	10
Group 11	Evito (fluoxastrobin)	140	269	1	1	1	1	2	3	1	1	4	4	3	2	2	2	2	3	6	6	4	4
	Flint (trifloxystrobin)	70	140	2	2	1	1	3	4	1	1	5	5	2	2	2	2	1	1	3	4	4	4
	Quadris (azoxystrobin)	1250	2500	5	7	3	4	2	3	4	4	7	7	3	4	4	5	2	2	6	6	6	8
Group 12	Medallion (fludioxonil)	276	552	7	7	8	9	4	5	8	9	8	7	8	8	8	7	8	8	9	9	9	10
Group 17	Elevate (fenhexamid)	850	1700	8	9	7	7	5	6	3	3	3	4	10	10	4	5	5	6	7	7	3	4
Group 19	OSO (polyoxin D)	11	38	5	5	9	9	2	2	6	7	4	5	6	7	4	3	2	4	9	9	6	6
Group 33	Aliette (fosetyl-AI)	4400	8800	10	10	10	10	10	10	10	10	10	10	10	10	10	10	10	10	10	10	10	10

The efficacy is presented as the percentage reduction in the mycelial growth by each fungicide in comparison to the mycelial growth in the absence of the fungicide, on a scale of 0 to 10, where 0 to 1 = no efficacy 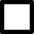, 2 to 4 = low efficacy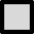, 5 to 7 = moderate efficacy 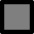, and 8 to 10 = high efficacy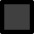.

*Inhibition of mycelial growth*: Petri plates containing ¼PDA medium amended with the minimum and maximum rates of each fungicide (**Table 1**) were prepared for the bioassay. Similarly prepared assay plates without the fungicides were used as a control. To assess the efficacy of fungicides to each of the ten pathogens, three assay plates at the minimum and maximum rates of each fungicide were used as replicates. Mycelial plugs of 5 mm diameter were taken from five- to seven-day-old actively growing cultures of the pathogens on PDA medium and were placed in the centre of the fungicide amended and non-amended (control) assay plates. Plates were incubated in the dark at the optimum growth temperature (20 to 24°C) for each pathogen as determined previously. Once the growth of the pathogen on the control plates almost reached the edge of the plates, two perpendicular measurements of the diameter of the fungal colonies were taken from each of the three replicate plates of fungicide amended and non-amended treatments, where the original diameter of 5 mm of the mycelial plug was subtracted from the measurements. Efficacy of the fungicide to inhibit the growth of mycelium of cranberry fruit rot pathogens was determined as a mean percentage reduction in the mycelial growth of the pathogen on fungicide amended medium compared to that of non-amended medium. The mean percentage reduction in the mycelial growth, i.e., percentage inhibition, was also presented on a scale of 0 to 10, where 0 = no inhibition, 1 = ≤ 10% inhibition, 2 = 10 - 20% inhibition, 3 = 20 - 30% inhibition, 4 = 30 - 40% inhibition, 5 = 40 - 50% inhibition, 6 = 50 - 60% inhibition, 7 = 60 - 70% inhibition, 8 = 70 - 80% inhibition, 9 = 80 - 90% inhibition and 10 = 90 - 100% inhibition.

*Inhibition of spore germination*: As described previously, fungicides were prepared at minimum and maximum concentrations in ¼PDA medium and placed in a water bath at 50°C. Volumes of 1 mL assay medium were then dispensed in sterile, twenty four-well cell culture plates (Falcon^®^) and allowed to solidify. A similar set of cell culture plates without the fungicides were used as control. For each treatment, sets of two similarly prepared micro-wells were served as replicates. Spores of either conidia or ascospores from the pathogens grown on either PDA or ¼PDA medium were harvested in sterile distilled water amended with 0.1% TWEEN^®^ 20 (Sigma-Aldrich) and the concentration was adjusted to 1 x 10^4^ spores ml^-1^ with an aid of a hemocytometer. Aliquots of 20 µl spore suspensions were placed onto the assay wells and the plates were incubated in the dark at 18°C for two to five days until the mycelial growth of the pathogen on the fungicide non-amended control wells was visible. Efficacy of the fungicides to inhibit the germination of spores of fruit-rot pathogens of cranberry was assessed based on the absence, i.e., positive (+) inhibition, or presence, i.e., negative (-) inhibition, of mycelial growth of the pathogen on fungicide-amended assay wells compared to the mycelial growth of the pathogen on fungicide non-amended assay wells.

### 2.3 Statistical analysis

Where applicable and with a few exceptions, analysis of variance (ANOVA) or Fisher exact test of 2 x 2 contingency table were performed in the comparison of mean percentage incidence of fruit rot pathogens in the flower, green and ripe fruit samples and mean percentage incidence of the fruit rot pathogens within cranberry producing regions, using the Statistical Software R and the user interface RStudio (R Core Team, 2016).

## 3 Results

### 3.1 Prevalence, incidence and spatial distribution of cranberry fruit rot fungi

#### 3.1.1 Prevalence of fruit rot fungi

**Figure 2:**
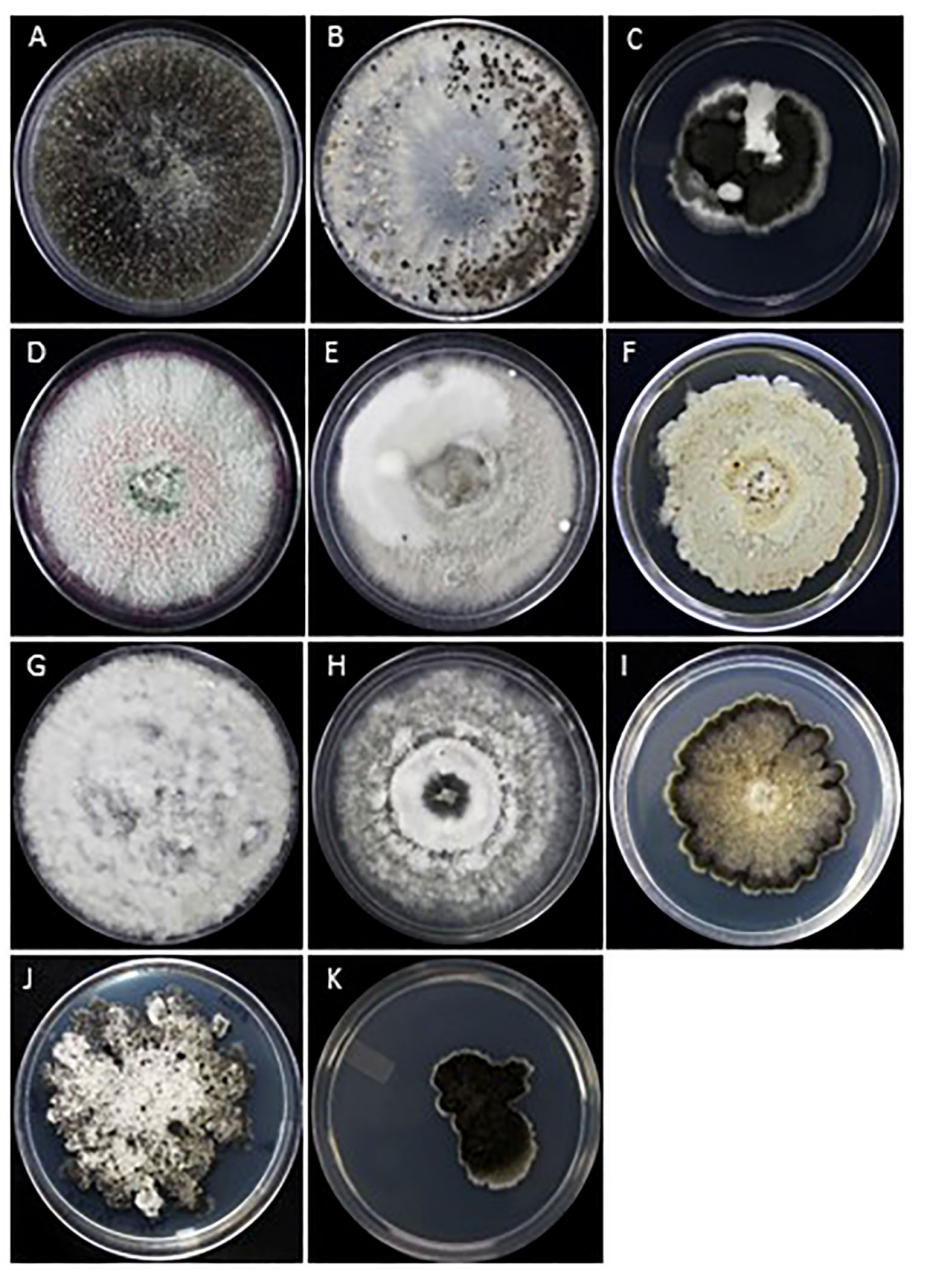
The fungal pathogens of cranberry fruit rot were isolated from flowers, green and ripe fruit samples collected from twenty-eight farms in southwestern British Colombia. Fungal cultures on PDA medium, **(A)** *Allantophomopsis lycopodina*, **(B)** *Botrytis cinerea*, **(C)** *Coleophoma empetri*, **(D)** *Colletotrichum fioriniae*, **(E)** *Colletotrichum gloeosporioides*, **(F)** *Fusicoccum putrefaciens*, **(G)** *Glomerella cingulata*, **(H)** *Phomopsis vaccinii*, **(I)** *Physalospora vaccinii*, **(J)** *Phyllosticta elongata*, and **(K)** *Phyllosticta vaccinii*.

The major fungal pathogens causing fruit rot of cranberry in British Columbia were identified from the samples of flowers, and green and ripe fruits collected during two consecutive crop seasons from twenty-eight fields, representing nearly 20% of the cranberry farms in southwestern British Columbia (**Figure 1**). Diseases of cranberry fruit caused by either bacteria or viruses, if any, were excluded in this study. Fungal pathogens found on the samples were successfully isolated at high frequencies using the a¼PDA medium compared to the three other fungal isolation media that were tested (data not presented). a¼PDA medium served as the most suitable medium for the isolation of a broad spectrum of fungi and aided in preventing any bacterial contamination originating from the cranberry tissues. Fungal isolates were identified based on colony growth characteristics and morphology of spores produced on either a¼PDA or PDA medium and further confirmed by PCR and DNA sequence analysis. The most frequently isolated fungi were *Allantophomopsis cytisporea*, *Botrytis cinerea*, *Coleophoma empetri*, *Colletotrichum fioriniae* (formally *C. acutatum*), *Colletotrichum gloeosporioides*, *Fusicoccum putrefaciens*, *Glomerella* sp., *Phomopsis vaccinii* (hereafter refer to as *Phom. vaccinii*), *Phyllosticta elongata* (hereafter refer to as *Phyl*. *elongata*), *Phyllosticta vaccinii* (hereafter refer to as *Phyl*. *vaccinii*) and *Physalospora vaccinii* (hereafter refer to as *Phys. vaccinii*) (**Figure 2**). Besides the commonly isolated fungi, species of *Alternaria*, *Aspergillus*, *Cladosporium*, *Epicoccum*, *Fusarium*, *Mucor*, *Penicillium*, *Pestalotiopsis*, and yeasts were also recovered from the samples of flowers, and green and ripe fruits at low frequencies (data not presented).

#### 3.1.2 Incidence of fruit rot fungi

**Figure 3:**
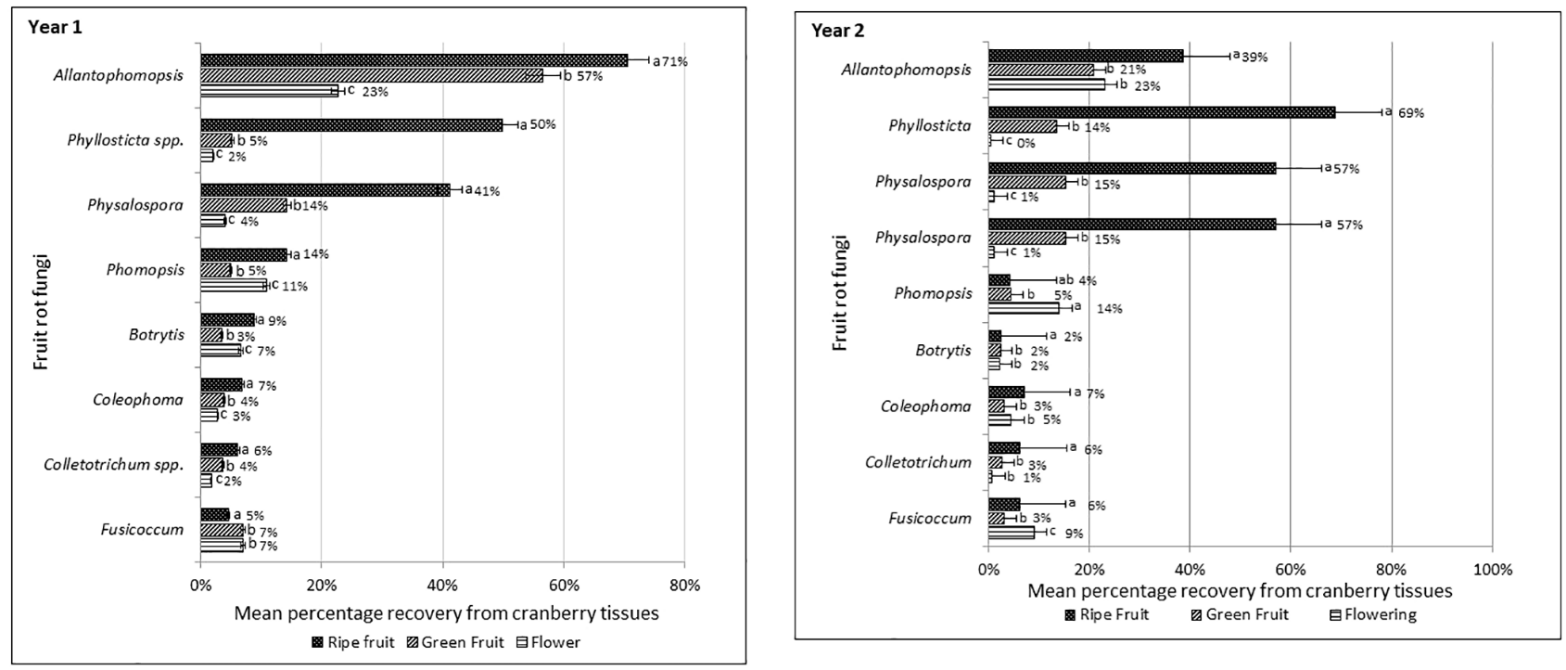
Mean percentage incidence of cranberry fruit rot pathogens recovered from the flowers, green and ripe fruit samples collected from fourteen farms in Year 1 and fourteen farms in Year 2 in southwestern British Columbia.

Incidence of fruit rot fungi in cranberry fields is presented as cumulative mean percentage incidence based on the percentage recovery of the fungal pathogens from the cranberry flowers, green and ripe fruit samples collected from twenty-eight farms during two consecutive crop seasons (**Figure 3**). In both years, regardless of the tissue type or phenological stages of the cranberry crop when samples were collected, the most frequently isolated fungi were *A. cytisporea*, *B. cinerea*, *C. empetri*, *Colletotrichum* spp. (hereafter, collectively referred to *C*. *fioriniae*, *C. gloeosporioides* and *Glomerella* sp.), *F. putrefaciens*, *Phom. vaccinii*, *Phyllosticta* spp. (hereafter, collectively referred to *Phyl*. *elongata* and *Phyl*. *vaccinii*) and *Phys. vaccinii*. The percentage incidence of the fruit rot fungi, based on their recovery from the samples, varied considerably amongst the flowers, and green and ripe fruits. In general, the percentage recovery of fruit rot fungi from ripe fruit was much greater than the percentage recovery from flowers or green fruit. In Year 1, the most frequently recovered fruit rot fungi from the ripe fruit samples were *A*. *cytisporea* (71%), *Phyllosticta* spp. (50%) and *Phys*. *vaccinii* (41%), followed by *Phom*. *vaccinii* (14%), *B.* cinerea (9%), *C*. *empetri* (7%), *Colletotrichum* spp. (6%) and *F*. *putrefaciens* (5%). A similar trend was observed in Year 2, where the most frequently recovered fungi from the ripe fruit samples were *Phyllosticta* spp. (59%), *A. cytisporea* (55%) and *Phys. vaccinii* (49%), whereas *Phom. vaccinii*, *C. empetri*, *Colletotrichum* spp., *B. cinerea* and *F. putrefaciens* were recovered at 9, 7, 6, 6 and 5%, respectively. The percentage recovery of fruit rot fungi from the flower and green-fruit samples varied amongst the samples and the farms from where they were collected. Overall, the most dominant fruit rot fungi recovered from the cranberry fields during two consecutive years were *A*. *cytisporea*, *Phyllosticta* spp. and *Phys*. *vaccinii*.

#### 3.1.3 Spatial distribution of cranberry fruit rot fungi

**Figure 4:**
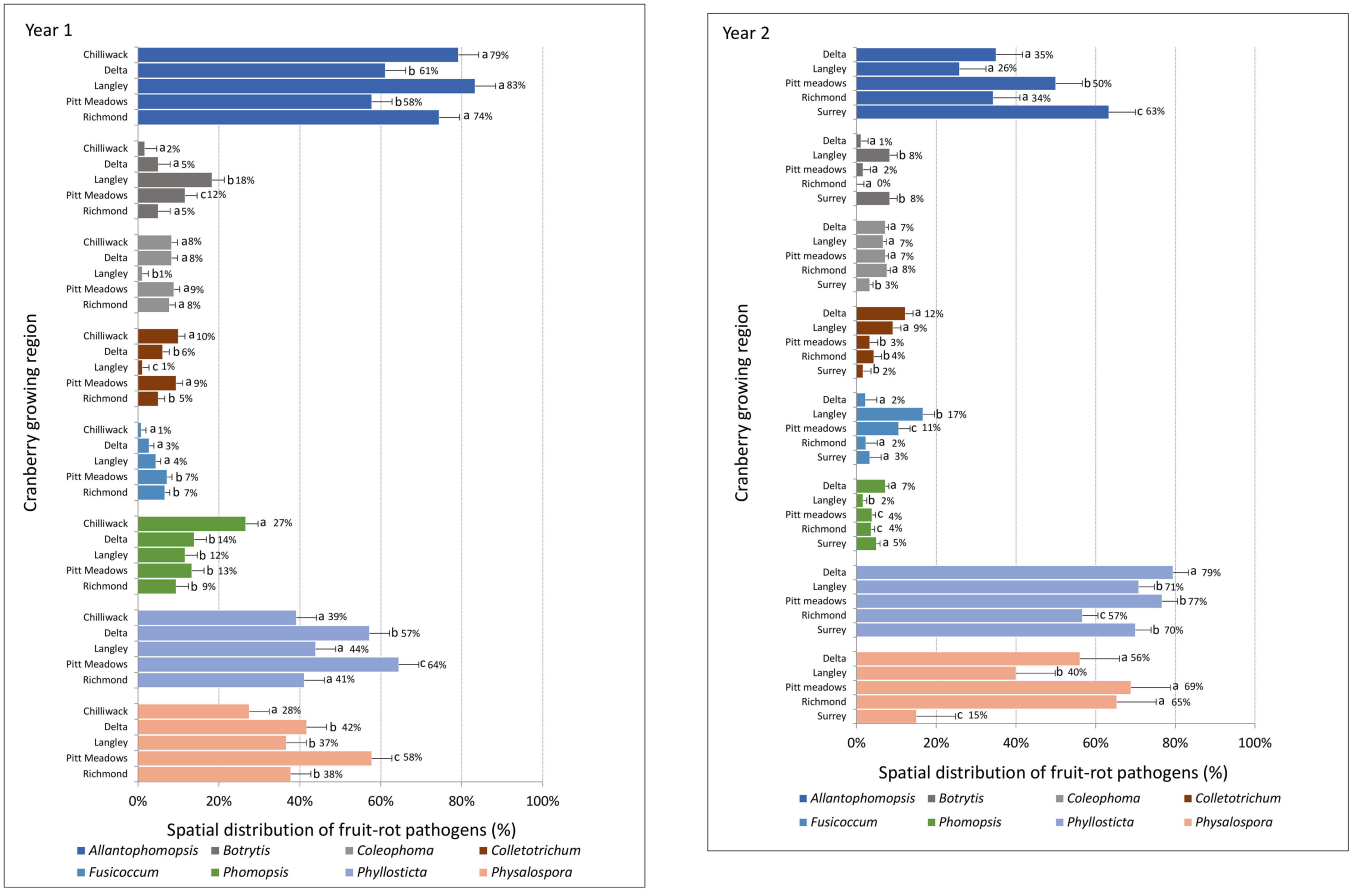
Mean percentage distribution of cranberry fruit rot pathogens as accounted from the ripe fruit samples collected at harvest from fourteen farms in Year 1 and fourteen farms in Year 2 in six cranberry growing regions in southwestern British Columbia.

**Figure 4** shows the spatial (geographical) distribution of fungal pathogens causing fruit rot in different cranberry producing regions of southwestern British Columbia as assessed based on the mean percentage of pathogens recovered from the samples of flowers, and green and ripe fruits collected from twenty-eight farms during two consecutive years. Although the number of farms sampled during two consecutive years varied from region-to-region and year-to-year, the prevalence of fruit rot pathogens in each region was evident from the percentage recovery of the pathogens from the samples at three phenological stages of the crop **Table 2**. In both years, *A*. *cytisporea*, *B*. *cinerea*, *C*. *empetri*, *C*. *fioriniae*, *C*. *gloeosporioides* and *Colletotrichum* spp., *F*. *putrefaciens*, *Phom*. *vaccinii*, *Phyllosticta* spp. and *Phys*. *Vaccinii* were found to be present in all cranberry producing regions in southwestern British Columbia. Although the number of farms sampled in each region was not consistent, *A*. *lycopodina*, *Phyllosticta* spp. and *Phys*. *vaccinii* were the most frequently recovered pathogens from all cranberry growing regions, particularly from the ripe fruit samples collected at harvest. In Year 1, the mean percentage recovery of *A*. *lycopodina* from the samples of ripe fruit from the farms in Chilliwack, Langley, Pitt Meadows, Richmond and Delta was 79, 83, 58, 74 and 61%, respectively, *Phyllosticta* spp. was 39, 44, 64, 41, and 57%, respectively, and *Phys*. *vaccinii* was 28, 37, 58, 38, and 42%, respectively. In Year 2, the mean percentage recovery of pathogens from the samples of ripe fruit from the farms in Langley, Surrey, Pitt Meadows, Richmond and Delta for *A*. *lycopodina* was 26, 63, 50, 34, and 35%, respectively, *Phyllosticta* spp. was 71, 70, 77, 57, and 79%, respectively, and *Phys*. *vaccinii* was 40, 15, 69, 65 and 56%, respectively. Although *B*. c*inerea*, *C*. *empetri*, *Colletotrichum* spp., *F*. *putrefaciens* and *Phom*. *vaccinii* were recovered from the samples of ripe fruit from all cranberry producing regions, the mean recovery percentages of these fungi were far less, i.e., between 1 and 27% in Year 1 and 1 and 17% in Year 2, than the mean recovery percentages of *A*. *lycopodina*, *Phyllosticta* spp. or *Phys*. *vaccinii*. Amongst the fruit rot pathogens, *A*. *lycopodina* and *F*. *putrefaciens* were detected at high frequencies as early as at flowering compared to other fruit rot pathogens.

**Table 2:** Spatial distribution of cranberry fruit rot pathogens, as assessed based on the mean percentage incidence of the pathogens on flowers, green and ripe fruit samples collected from twenty-eight farms during two consecutive years, is presented as cumulative percentage incidence in six cranberry growing regions in southwestern British Columbia.

Cranberry Sample	Cumulative percentage incidence of fruit rot fungi											
	Chilliwack		Langley		Surrey		Pitt Meadows		Richmond		Delta	
	Year 1	Year 2	Year 1	Year 2	Year 1	Year 2	Year 1	Year 2	Year 1	Year 2	Year 1	Year 2
Allantophomopsis												
Flowers	42	–	24	37	–	7	24	16	23	13	7	43
Green fruit	80	–	67	13	–	13	46	19	49	17	49	37
Ripe fruit	79	–	83	26	–	63	58	50	74	34	61	35
Botrytis												
Flowers	3	–	9	5	–	0	10	4	5	0	6	2
Green fruit	1	–	3	2	–	0	9	1	3	2	1	6
Ripe fruit	2	–	18	8	–	8	12	2	5	0	5	1
Coleophoma												
Flowers	3	–	1	6	–	0	1	1	7	4	2	11
Green fruit	2	–	3	4	–	2	7	3	4	4	3	1
Ripe fruit	8	–	1	7	–	3	9	7	8	8	8	7
Colletotrichum spp.												
Flowers	1	–	1	0	–	0	3	0	1	2	4	0
Green fruit	10	–	0	1	–	5	4	1	3	5	4	1
Ripe fruit	10	–	1	9	–	2	9	3	5	4	6	12
Fusicoccum												
Flowers	0	–	8	20	–	0	7	23	16	3	3	1
Green fruit	3	–	11	10	–	0	8	7	9	1	3	0
Ripe fruit	1	–	4	17	–	3	7	11	7	2	3	2
Phomopsis												
Flowers	9	–	4	3	–	13	16	3	23	19	4	24
Green fruit	14	–	2	5	–	8	6	6	2	4	4	3
Ripe fruit	27	–	12	2	–	5	13	4	9	4	14	7
Phyllosticta spp.												
Flowers	5	–	4	0	–	0	0	1	1	0	1	1
Green fruit	4	–	7	8	–	3	11	18	3	15	1	15
Ripe fruit	39	–	44	71	–	70	64	77	41	57	57	79
Physalospora												
Flowers	0	–	11	0	–	0	4	0	1	3	3	0
Green fruit	8	–	8	5	–	0	24	33	9	16	20	9
Ripe fruit	28	-	37	40	–	15	58	69	38	65	42	56

#### 3.1.4 Assessment of fruit rot incidence

The mean percentage incidence of fruit rot from the samples of ripe fruit collected at harvest and a 3-week post-harvest storage at ambient temperature from fourteen farms in Year 1 and fourteen farms in Year 2 is shown in **Table 3**. In both years, the mean percentage incidence of fruit rot at harvest and at three weeks of post-harvest storage varied amongst the farms and geographical regions. In Year 1, the highest incidence of fruit rot at harvest was recorded at 24% in Chilliwack (Farm 1) followed by 16% fruit rot in Delta (Farm 1), 8% fruit rot in Pitt Meadows (Farm 1), and 7% fruit rot in a farm in Richmond (Farm 3). The incidence of fruit rot at harvest was ≤ 5% in nine out of fourteen farms sampled. In Year 2, the highest incidence of fruit rot at harvest was recorded at 18% in Delta (Farm 3), followed by 10, 8 and 7% fruit rot in Richmond (Farms 1, 5 and 4, respectively), and 9% fruit rot in Delta (Farm 2). Overall, the incidence of fruit rot was < 5% in nine of the fourteen farms sampled in the second year. In both years, the mean percentage incidence of fruit rot increased considerably when symptomless ‘healthy’ fruit samples were stored for three weeks at ambient temperature (**Table 3**). In Year 1, the mean percentage incidence of fruit rot at three-week of post-harvest storage of the samples from the fourteen farms ranged from 11 to 61%. Over 40% of the post-harvest rot was recorded in four farms (Chilliwack Farm 1, Langley Farm 1, Pitt Meadows Farm 1 and 3), 20 to 40% fruit rot in 6 farms (Chilliwack Farm 2, Langley Farm 2, Pitt Meadows Farm 2, Richmond Farm 1 and 2, Delta Farm 1), and < 20% fruit-rot in four farms (Langley Farm 3, Richmond Farm 3, Delta Farm 2 and 3). Amongst the fourteen farms sampled in Year 1, two of the farms in Pitt Meadows (Farm 3 and 1) and one of the farms in Chilliwack (Farm 1) had the highest percentage of post-harvest rot, accounting for 61, 56 and 49%, respectively. A similar trend was observed in Year 2, where the mean percentage incidence of post-harvest rot ranged between 7 and 67%. A high incidence of fruit rot was accounted for on the farms in Richmond (21 to 67%), Pitt Meadows (44 to 53%) and Delta (33 to 53%). Amongst the fourteen farms sampled in Year 2, six (Pitt Meadows 1, 2 and 3, Richmond 1 and 4, Delta 3) had > 40% post-harvest rot, six (Langley 2, Richmond 2, 3, and 5, Delta 1 and 2) had 20 to 40% post-harvest rot, and two (Surrey 1 and Langley 1) had < 20% of post-harvest rot.

**Table 3:** Mean percentage incidence of fruit rot of ripe fruit samples, collected from fourteen cranberry farms in Year 1 and fourteen cranberry farms in Year 2, at harvest and at a 3-week post-harvest storage at ambient temperature.

Year 1 - Mean percentage fruit rot incidence				Year 2 - Mean percentage fruit rot incidence			
Farm Location	At harvest	At 3-week storage	Cumulative	Farm Location	At harvest	At 3-week storage	Cumulative
Chilliwack				Surrey			
Farm 1	24	49	73	Farm 1	1	18	19
Farm 2	3	20	23				
Langley				Langley			
Farm 1	3	43	46	Farm 1	3	7	10
Farm 2	5	24	29	Farm 2	1	20	21
Farm 3	1	13	14				
Pitt Meadows				Pitt Meadows			
Farm 1	8	56	64	Farm 1	3	53	56
Farm 2	7	24	31	Farm 2	2	42	44
Farm 3	2	61	63	Farm 3	1	44	45
Richmond				Richmond			
Farm 1	3	26	29	Farm 1	10	67	77
Farm 2	4	28	32	Farm 2	0	38	38
Farm 3	7	11	18	Farm 3	1	27	28
				Farm 4	7	58	65
				Farm 5	8	21	29
Delta				Delta			
Farm 1	16	20	36	Farm 1	1	33	34
Farm 2	0	17	17	Farm 2	9	32	41
Farm 3	3	19	22	Farm 3	18	53	71

Mean percentage incidence of fruit rot in each farm was calculated from three replicate samples, with a sample size of 40 fruit per replicate, thus a total of 120 fruit.

#### 3.1.5 Efficacy of fungicides to fruit rot pathogens

Twenty six chemical fungicides belonging to nine different modes of actions (FRAC groups) tested at the minimum and maximum rates as per manufacturer’s label for the efficacy to inhibit the mycelial growth and germination of spores of fruit rot pathogens are presented as the mean percentage reduction of mycelial growth (Scale of 1 to 10), and presence or absence of spore germination in **Tables 1**, **4**, respectively.

**Table 4:** Twenty-six chemical fungicides, belonging to 9 different groups of modes of actions (FRAC Code List, 2022), were evaluated at the minimum and maximum application rates for the efficacy to inhibit the spore germination of cranberry fruit rot pathogens.

Inhibition of spore germination of cranberry fruit-rot pathogens by fungicides											
Fungicide (active ingredient)		Fruit rot pathogens									
		*A. lycopodina*	*B. cinerea*	*C. empetri*	*C. acutatum*	*C. gloeosporioides*	*F. putrefaciens*	*G. cingulata*	*Phom. vaccinii*	*Phyl. elongata*	*Phys. vaccinii*
Group M	Bravo (chlorothalonil)										
	Copper 53W (copper sulfate)										
	Cueva (copper octanoate)										
	Guardsman (copper oxychloride)										
	Kocide (copper hydroxide)										
	Maestro (captan)										
Group 3	Fullback (flutriafol)										
	Funginex (triforine)										
	Indar (fenbuconazole)										
	Inspire (difenoconazole)										
	Proline (prothioconazole)										
	Tilt (propiconazole)										
Group 7	Adepidyn (pydiflumetofen)										
	Aprovia (benzovindiflupyr)										
	Fontelis (penthiopyrad)										
	Kenja (isofetamid)										
	Sercadis (fluxapyroxad)										
Group 9	Scala (pyrimethanil)										
	Vangard (cyprodinil)										
Group 11	Evito (fluoxastrobin)										
	Flint (trifloxystrobin)										
	Quadris (azoxystrobin)										
Group 12	Medallion (fludioxonil)										
Group 17	Elevate (fenhexamid)										
Group 19	OSO (polyoxin D)										
Group 33	Aliette (fosetyl-AI)										

The efficacy is presented as inhibition of spore germination at the maximum and minimum rates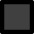, maximum rate only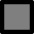, no inhibition at maximum and minimum rates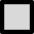, and no data is available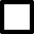.

*Inhibition of mycelial growth*: Group M Fungicides Copper 53W (copper sulfate), Cueva (copper octanoate), Kocide (copper hydroxide) and Maestro 80DF (captan) were highly effective (scale 8 to 10) at inhibiting the mycelial growth of all fungal pathogens. Although fungicides Bravo (chlorothalonil) and Guardsman (copper oxychloride) were highly effective (scale 8 to 10) against most fungal pathogens, Bravo was moderately effective (scale 5 to 7) against *B*. *cinerea* and *C*. *empetri* and less effective (scale 2 to 4) against *F*. *putrefaciens*, and Guardsman was moderately effective (scale 5 to 7) against *B*. *cinerea* and less effective (scale 2 to 4) against *F*. *putrefaciens*. Amongst the Group 3 fungicides, Fullback (flutriafol), Funginex (triforine), Inspire (difenoconazole), Proline (prothioconazole) and Tilt (propiconazole) were highly effective (scale 8 to 10) at inhibiting the mycelial growth of all fungal pathogens, with the exception of Indar (fenbuconazole), being highly effective against most fungal pathogens but moderately effective (scale 5 to 7) at inhibiting the mycelial growth of *C*. *gloeosporioides*, *G*. *cingulata* and *Phys. vaccinii*. Within the group 7 fungicides, only Aprovia (benzovindiflupyr) was highly effective (scale 8 to 10) at inhibiting the mycelial growth of all fungal pathogens. Adepidyn (pydiflumetofen), with the exception of *Phyl*. *elongata*, was moderate (scale 5 to 7) to less (scale 2 to 4) effective at inhibiting the mycelial growth of fruit rot pathogens. Fontelis (penthiopyrad) was highly effective (scale 8 to 10) against *G*. *cingulata*, but it was less (scale 2 to 4) or moderately (scale 5 to 7) effective against all other cranberry fruit rot pathogens. Kenja (isofetamid) and Sercadis (fluxapyroxad) were moderately effective (scale 5 to 7) at inhibiting the mycelial growth of *B*. *cinerea* and *Phyl*. *elongata* but had less (scale 2 to 4) to no effect on all other fruit-rot pathogens. Of the two group 9 fungicides tested, Scala (pyrimethanil) was highly effective (scale 8 to 10) at inhibiting the mycelial growth of most cranberry fruit rot pathogens but was moderately effective (scale 5 to 7) at inhibiting the mycelial growth of *C*. *acutatum* and *C*. *gloeosporioides*, whereas Vangard (cyprodinil) was highly effective (scale 8 to 10) against *A*. *lycopodina*, *B. cinerea*, *F*. *putrefaciens*, *Phom. vaccinii*, *Phyl*. *elongata* and *Phys*. *vaccinii* and moderately (scale 5 to 7) effective against *C*. *acutatum* and *C*. *gloeosporioides*. In general, group 11 fungicides Flint (trifloxystrobin), Evito (fluoxastrobin) and Quadris (azoxystrobin) were less (scale 2 to 4) to moderately effective (scale 5 to 7) against the fruit rot pathogens. Quadris was moderately effective (scale 5 to 7) against *A*. *lycopodina*, *C*. *gloeosporioides*, *Phyl*. *elongata* and *Phys*. *vaccinii*, and Flint and Evito were moderately effective (scale 5 to 7) against *C*. *gloeosporioides* and *Phyl*. *elongata*, respectively, but had less (scale 2 to 4) or no activity against other fruit rot pathogens. Group 12 fungicide Medallion (fludioxonil) was highly effective (scale 8 to 10) at inhibiting the mycelial growth of most cranberry fruit rot pathogens, but it was moderately effective (scale 5 to 7) against *A*. *lycopodina* and *C*. *empetri*. Elevate (fenhexamid), a group 17 fungicide, was highly effective (scale 8 to 10) at inhibiting the mycelial growth of *A*. *lycopodina* and *F*. *putrefaciens* but was less (scale 2 to 4) or moderately (scale 5 to 7) effective against all other fruit rot pathogens. Group 19 fungicide Polyoxin D (OSO) was highly effective (scale 8 to 10) at inhibiting the mycelial growth of *B*. *cinerea* and *Phyl*. *elongata* but was less (scale 2 to 4) to moderately (scale 5 to 7) effective at inhibiting the mycelial growth of all other fungal pathogens. Group 33 fungicide Aliette (fosetyl-Al) was highly effective (scale 8 to 10) at inhibiting the mycelial growth of all cranberry fruit rot pathogens.

*Inhibition of spore germination*: In general, group M fungicides Bravo, Copper 53W, Cueva, Kocide and Maestro 80DF were effective at inhibiting the spore germination of fruit rot pathogens at both maximum and minimum rates, with the exception of Bravo and Kocide that had no effect on the spore gemination of *B*. *cinerea*. Guardsman did not prevent the spore germination of cranberry fruit rot pathogens, except *C*. *gloeosporioides* at the maximum rate. Amongst the group 3 fungicides, Fullback, Funginex, Inspire, Proline and Tilt inhibited the spore germination of fruit rot pathogens at both maximum and minimum rates, with the exception of Fullback and Tilt that had no effect on *B*. *cinerea*. Indar was effective at inhibiting the spore germination of most cranberry fruit rot pathogens, but it had no activity against *C*. *acutatum*, *C*. *gloeosporioides*, *G*. *cingulata*, and *Phom vaccinii*. Within the group 7 fungicides, Aprovia was highly effective at inhibiting the spore germination of fruit rot pathogens at both maximum and minimum rates whereas Adepidyn and Kenja were only effective against *Phyl*. *elongata*, and Fontelis was effective against *C*. *gloeosporioides* and *Phyl*. *elongata*. Sercadis had no effect on the spore germination of any of the fruit rot pathogens. Within the group 9 fungicides, Scala was ineffective at inhibiting the spore germination of *C*. *acutatum*, *C*. *gloeosporioides* and *Phom*. *vaccinii* but was effective against other fruit rot pathogens, whereas Vangard was effective against *A*. *lycopodina*, *F*. *putrefaciens*, and *Phyl*. *elongata* and had no activity against other fruit rot pathogens. In general, Group 11 fungicides Evito, Quadris and Flint were ineffective at inhibiting the spore germination of fruit rot pathogens with the exception of Flint that had inhibitory activity against *A*. *lycopodina*. Group 12 fungicide Medallion was effective at inhibiting the spore germination of *A*. *lycopodina* and *Phyl*. *elongata* but was ineffective against all other fruit rot pathogens. Elevate, group 17, was ineffective at inhibiting the spore germination of cranberry fruit rot pathogens with the exception of *B*. *cinerea* at the maximum rate. Polyoxin D, a group 19 fungicide, was ineffective at inhibiting the spore germination of cranberry fruit rot pathogens whereas Aliette, a group 33 fungicide, was effective at inhibiting the spore germination of all cranberry fruit rot pathogens.

## 4 Discussion

Pre- and post-harvest fruit loss due to fruit rot diseases of cranberry caused by fungal pathogens is a common occurrence in British Columbia. Cranberry growers have been employing management strategies, both cultural practices and application of fungicides, to minimize fruit loss and maintain fruit quality. However, the knowledge of types of fruit rot pathogens present, and their distribution, incidence and extent of impact on cranberry production in different regions in British Columbia has been limited. The lack of such information on fruit rot pathogens is an impediment to the development or employment of effective management strategies to combat the pathogens that contribute to yield loss and poor fruit quality. This study provides a comprehensive analysis of fungal pathogens that are responsible for cranberry fruit rot complex. Samples were collected for analysis at three phenological stages of the cranberry crop, i.e., flowers, and green and ripe fruits, from 28 farms over a two-year period in six major cranberry growing regions in southwestern (Lower Mainland) British Columbia.

It is apparent from this study that at least eleven fungal pathogens, *Allantophomopsis cytisporea* (to a lesser extent *A*. *lycopodina*), *B*. *cinerea*, *C*. *empetri*, *Colletotrichum* spp. (*C*. *acutatum* and *C*. *gloeosporioides*), *F*. *putrefaciens*, *Glomerella* sp., *Phom*. *vaccinii*, *Phyllosticta* spp. (*Phyl*. *vaccinii* and *Phyl*. *elongata*) and *Phys*. *vaccinii* contribute to fruit rot diseases of cranberry in British Columbia. These pathogens have been previously reported to cause pre- and post-harvest fruit rot in other cranberry production regions in North America and elsewhere (Polashock et al., 2009; Wells and McManus, 2013; Polashock et al., 2017; Wells-Hansen and McManus, 2017; Conti et al., 2022). Based on a limited studies and anecdotal evidence, the fungal pathogens *Allantophomopsis* spp. (*A*. *lycopodina*, and *A*. *cytisporea*), *B*. *cinerea*, *C*. *empetri*, *F*. *putrefaciens*, *G*. *cingulata*, *Monilinia oxycocci*, *Phom*. *vaccinii*, *Phyl*. *elongata*, *Phys*. *vaccinii*, and *Strasseria geniculata* have been considered as possible causes of pre- and post-harvest fruit rot of cranberry in the Pacific Northwest (Strik et al., 2002). However, there is no verification on the qualitative and quantitative nature of these pathogens in the Pacific Northwest. A small-scale survey conducted by Caruso in 2015/2016 confirmed the occurrence of some of the critical fruit rot pathogens in the Pacific Northwest (personal communication).

Although the percentage recovery of fruit rot pathogens varied between cranberry samples, the fungal pathogens, as identified in this study, were consistently recovered from the flower, and green and ripe fruit samples. Detection of these fungal pathogens, regardless of where the samples were collected from, shows that these pathogens were prevalent in all cranberry producing regions in southwestern British Columbia. Furthermore, the spatial distribution of each pathogen appeared to be not associated with the scale (hectares) of production or climatic conditions between the regions. In British Columbia, cranberry has been commercially cultivated since as early as 1940. Therefore, the production practices over the years may have contributed to the introduction and movement of major fruit rot pathogens across the cranberry growing regions. Today, the region of Richmond has the highest acreage (≈40%) of cranberry production, followed by Delta (≈30%), Pitt Meadows (≈10%), Langley (≈5%), Surrey (≈3%) and Chilliwack (≈2%). Local farmers have been using propagation material exclusively brought in from northeastern United States, and, to a lesser extent, from vine cuttings obtained locally from established ‘healthy’ fields. As a result, the movement of propagation material from field-to-field and region-to-region may have contributed to the spread of the pathogens across the cranberry production regions in British Columbia.

Based on the percentage recovery of fruit rot pathogens from the flowers, and green fruit and ripe fruit samples collected from twenty-eight farms over a 2-year period, *A*. *cytisporea* (black rot), *Phyl*. *elongata* (early rot) and *Phyl*. *vaccinii* (berry speckle) and *Phys*. *vaccinii* (blotch rot) have been identified as the most prevalent pathogens causing fruit rot of cranberry in British Columbia. Depending on the field and location, the percentage recovery from the ripe fruit samples for *A*. *cytisporea* was in the range of 26 to 83%, *Phyllosticta* spp. were in the range of 39 to 79%, and *Phys*. *vaccinii* was in the range of 15 to 69%. Although pathogens *B*. *cinerea*, *C*. *empetri*, *Colletotrichum* spp., *F*. *putrefaciens* and *Phom*. *vaccinii* were frequently recovered from the ripe fruit samples, depending on the pathogen, their percentage recovery was much less, from 1 to 27%, than the three major fruit rot pathogens. This suggest that *A*. *cytisporea*, *Phyllosticta* spp. and *Phys*. *vaccinii* can be considered as the major contributors to the pre- and post-harvest fruit losses in British Columbia. A 3-year study conducted in Michigan demonstrated that *C*. *acutatum*, *Pestalotia vaccinii* and *Phyl*. *vaccinii* were predominant on rotted cranberry fruit compared to *A*. *lycopodina*, *Phys*. *vaccinii*, *C*. *empetri* or *B*. *cinerea* (Olatinwo et al., 2003). In Massachusetts and New Jersey, *Phyllosticta* spp. (*Phyl*. *elongata* and *Phyl. vaccinii*), *C*. *empetri*, *Phys*. *vaccinii*, and *Colletotrichum* spp. were identified as the major causative agents of cranberry fruit rot (Shear et al., 1931; Bergman and Wilcox, 1936; Caruso and Ramsdell, 1995; Stiles and Oudemans, 1999). In Québec, *A*. *cytisporea*, *C*. *empetri*, *Godronia cassandrae*, and *C*. *fructivorum* were more predominantly encountered than other fungi in cranberry farms (Conti et al., 2022). These observations indicate that there are regional differences in the prevalence and spatial distribution of fruit rot pathogens in cranberry fields, particularly between the northwestern and northeastern cranberry producing regions in North America. The demonstrated differences in the prevalence and distribution of fruit rot pathogens amongst different cranberry producing regions should be taken into consideration when strategizing and employing management strategies to target specific pathogens that are specific to each region and, thereby, minimize their impact on fruit quality and yield. Besides, based on the recovery of fruit rot pathogens from the cranberry samples collected at different phenological stages of cranberry crops, it is apparent that the pathogens can be detected as early as at flowering and green fruit stages of the crop. Although the frequency of recovery of the pathogens from flowers and green fruit was far less than the ripe fruit samples, the incidence of pathogens on flowers and green fruit supports the findings of their early season infection and latency in flowers and green fruit (Polashock et al., 2017). This emphasizes the importance of employing appropriate disease prevention strategies as early as at flowering to prevent the early infection and subsequent disease development by fruit rot pathogens.

Apart from the major fruit rot fungi, species of *Alternaria*, *Aspergillus*, *Cladosporium, Epicoccum*, *Fusarium*, *Muco*r, *Penicillium*, *Pestalotiopsis*, and yeasts were also recovered from the flowers, and green and ripe fruit samples. Although these fungi were detected at low frequencies, they have not been implicated in causing pre- or post-harvest fruit rot on cranberries. However, they could be regarded as opportunistic saprophytes, contributing synergistically to post-harvest and storage-rot of cranberries. Evidently, some of these fungi have been known to cause post-harvest rot of several other fruit commodities such as berries, stone fruit, and others.

The mean percentage fruit losses due to fruit rot as estimated from the samples of ripe fruit collected at harvest varied considerably amongst the fields and regions in both Year 1 and Year 2. The mean percentage of fruit losses in cranberry fields at harvest were found to be between 0 and 24% in Year 1 and 0 to 18% in Year 2. As expected, many factors could contribute to the observed range in the percentage fruit rot incidence from region-to-region, field-to-field and year-to-year. Such factors can be the result of differences in the occurrence and distribution of the type of fruit rot pathogens and levels of inoculum present in the fields, macro and micro climatic conditions, and overall cultural and disease management practices implemented, including selection of fungicides, timing and application efficiency of fungicides. The incidence of fruit rot after three-weeks of post-harvest storage at an ambient temperature was estimated to be from 4% at harvest to 50% after storage. This indicates that any delay in fruit harvest, i.e., leaving over-ripe fruit in the field or holding harvested fruit at the farm or at receiving or processing facilities without adequate controlled environmental storage conditions, could result in an increased incidence of post-harvest fruit spoilage. In this study, it is important to recognize that the estimated fruit losses could also be caused by both biotic and abiotic factors since, in some cases, it was not feasible to visually differentiate ‘unhealthy’ fruit based on the symptoms as a result of fungal pathogens or abiotic factors.

Pre- and post-harvest fruit rot of cranberry is caused by several fungal pathogens, which complicates management because control measures must be effective for multiple pathogens. The number of fungicides that are currently available to control the cranberry fruit rot complex in British Columbia is limited and may not have the efficacy to control the pathogens of interest. These pathogens vary in their biology, virulence, time of infection, and rate of spread, and are often dictated by their population densities and climatic conditions that vary from year-to-year, farm-to-farm and region-to-region. Therefore, farmers are expected to pay close attention when selecting fungicides and timing their application to maximize fruit rot control. To address the need for the efficacy of fungicides on fruit rot pathogens, a total of twenty six fungicides belonging to nine different modes of actions (FRAC groups) were assessed in this study. The fungicides displayed varying degrees of inhibitory activity against the mycelial growth and spore germination of the pathogens under laboratory conditions. In general, the fungicides Copper 53 (copper sulphate), Cueva (copper octonoate), Kocide (copper hydroxide) and Maestro (captan) of group M, Fullback (flutriafol), Funginex (triforine), Inspire (difenoconazole), Proline (prothioconazole) and Tilt (propiconazole) of group 3, Aprovia (benzovindiflupyr) of group 7, and Aliette (fosetyl-Al) of group 33 demonstrated a high degree of efficacy in inhibiting the mycelial growth of all pathogens. The fungicides Bravo (chlorothalonil) and Guardsman (copper oxychloride) of group M, Indar (fenbuconazole) of group 3, Scala (pyrimethanil) and Vangard (cyprodinil) of group 9, and Medallion (fludioxonil) of group 12 were effective against most the pathogens. Besides, fungicides Bravo (chlorothalonil), Copper 53W (copper sulfate), Cueva (copper octanoate) and Maestro (captan) of group M, Fullback (flutriafol), Funginex (triforine), Inspire (difenoconazole), Proline (prothioconazole) and Tilt (propiconazole) of group 3, Aprovia (benzovindiflupyr) of group 7 and Aliette (fosetyl-Al) of group 33 also effectively prevented the spore germination of most pathogens, hence these fungicides have the potential to minimize infection if applied preventatively prior to the onset of disease. In the *in vitro* assay, the group 11 fungicides Evito (fluoxastrobin), Flint (trifloxystrobin) and Quadris (azoxystrobin) demonstrated reduced efficacy to the cranberry fruit rot pathogens although azoxystrobin has been widely used to control the pathogens in commercial fields in the USA and Canada. This reduced efficacy could be attributed to the possibility of resistance development in the pathogens to the fungicide or its efficacy is only specific to certain pathogens but not all that are associated with the cranberry fruit rot complex. Therefore, further research is required to understand the efficacy of Group 11 fungicides to each of the pathogens present in different cranberry growing regions in North America. Since, in this study, the fungicide efficacy was tested *in vitro*, these fungicides need to be evaluated *in planta* to confirm their efficacy to control fruit rot diseases under field conditions. Besides, data on minimum residue level (MRL), toxicology on farm worker safety, and environmental impacts need to be collected in order to meet the environmental safety standards and to obtain official registration status from Health Canada. Furthermore, in British Columbia, fungicides are applied via the irrigation system (chemigation) which also requires specific environmental safety standards.

It is apparent from this study that cranberry fruit rot complex is caused by at least eleven fungal pathogens that have been present in all cranberry growing regions in southwestern (Lower Mainland) British Columbia. Therefore, farmers have to be proactive in identifying the pathogens present in fields and, therefore, apply efficient best management practices to minimize their impact on fruit quality and yield. The history of pathogens present at a farm and their disease epidemiology will dictate the selection of effective fungicides. Furthermore, approaches such as using appropriate timing and rates of fungicide application and implementing resistance management strategies, including rotating fungicides from different chemical groups and limiting the number of applications of fungicides that are medium to high risk for developing resistance in pathogens, will minimize the onset and development of fruit rot diseases of cranberry.

## Data availability statement

The original contributions presented in the study are included in the article/supplementary material. Further inquiries can be directed to the corresponding author.

## Author contributions

SS: Conceptualization, Funding acquisition, Methodology, Project administration, Resources, Supervision, Validation, Writing – original draft, Writing – review & editing. BW: Data curation, Formal Analysis, Investigation, Software, Writing – review & editing. EM: Data curation, Formal Analysis, Investigation, Software, Writing – review & editing. KN: Investigation, Resources, Writing – review & editing. TG: Investigation, Resources, Writing – review & editing.
